# Ferrihydrite Reduction by Photosynthetic *Synechocystis* sp. PCC 6803 and Its Correlation With Electricity Generation

**DOI:** 10.3389/fmicb.2021.650832

**Published:** 2021-03-08

**Authors:** Kenya Tanaka, Ginga Shimakawa, Shoko Kusama, Takashi Harada, Souichiro Kato, Shuji Nakanishi

**Affiliations:** ^1^Graduate School of Engineering Science, Osaka University, Toyonaka, Japan; ^2^Research Center for Solar Energy Chemistry, Osaka University, Toyonaka, Japan; ^3^Bioproduction Research Institute, National Institute of Advanced Industrial Science and Technology, Sapporo, Japan

**Keywords:** cyanobacteria, iron redox cycle, biophotovoltaics, extracellular electron transfer, *Synechocystis*

## Abstract

Microbial extracellular electron transfer (EET) to solid-state electron acceptors such as anodes and metal oxides, which was originally identified in dissimilatory metal-reducing bacteria, is a key process in microbial electricity generation and the biogeochemical cycling of metals. Although it is now known that photosynthetic microorganisms can also generate (photo)currents via EET, which has attracted much interest in the field of biophotovoltaics, little is known about the reduction of metal (hydr)oxides via photosynthetic microbial EET. The present work quantitatively assessed the reduction of ferrihydrite in conjunction with the EET of the photosynthetic microbe *Synechocystis* sp. PCC 6803. Microbial reduction of ferrihydrite was found to be initiated in response to light but proceeded at higher rates when exogenous glucose was added, even under dark conditions. These results indicate that current generation from *Synechocystis* cells does not always need light irradiation. The qualitative trends exhibited by the ferrihydrite reduction rates under various conditions showed significant correlation with those of the microbial currents. Notably, the maximum concentration of Fe(II) generated by the cyanobacterial cells under dark conditions in the presence of glucose was comparable to the levels observed in the photic layers of Fe-rich microbial mats.

## Introduction

**FIGURE 1:**
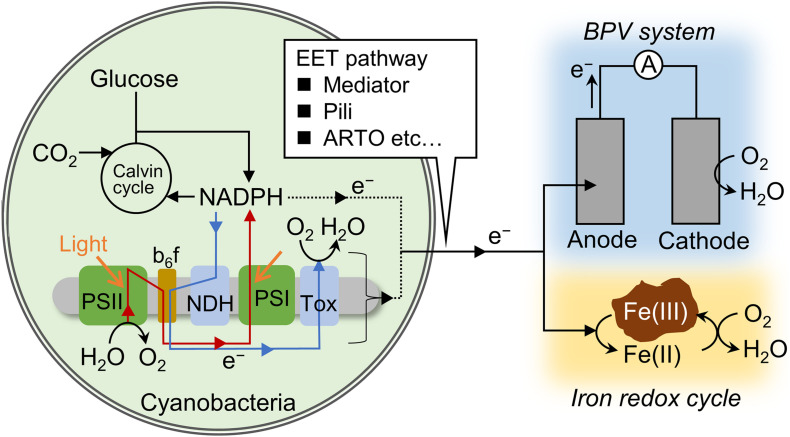
Schematic of the electron flux pathways related to anodic current generation and iron reduction. Red and blue arrows indicate photosynthetic and respiratory electron transfer chain. In photosynthetic electron transfer chain, electrons are transported from photosystem (PS) II to I *via* the interchain components, including plastoquinone, the cytochrome *b*_6_/*f* complex, and plastocyanin (or cytochrome *c*). In respiratory electron transfer chain, electrons are transported from NAD(P)H dehydrogenase (NDH) or succinate dehydrogenase to terminal oxidases (Tox) *via* the interchain components. Redox mediators, Type IV pili, and alternate respiratory terminal oxidase (ARTO) are suggested to function at the step of extracellular electron transfer (EET).

Photosynthetic microorganisms are able to transport intracellular electrons to an anode and this process, known as extracellular electron transfer (EET), has received attention within the field of biophotovoltaics (BPVs), as summarized in recent reviews (Rosenbaum et al., 2010; McCormick et al., 2015; Tschörtner et al., 2019). In a typical BPV system, oxygenic photosynthetic microorganisms such as cyanobacteria and green algae serve both as light absorbers and living anodes/catalysts for water oxidation. Previous researches have suggested that electrons are transferred from NADPH and/or electron transfer chain via some electron mediators, and/or Type IV pili (Lamb et al., 2014; Saper et al., 2018; Zhang et al., 2018). In cyanobacteria, due to the lack of organelles, both respiration and photosynthesis proceed in one cell, and they depend mutually in the cytosolic metabolism and in the electron transfer chain in the thylakoid membrane (Figure 1). Photosynthetic and respiratory electron transfer chains share the interchain components plastoquinone, the cytochrome *b*_6_/*f* complex, plastocyanin, and cytochrome *c* (Binder, 1982; Peschek et al., 2004). Therefore, not only photosynthetic reactions but respiratory reactions can affect EET flux. The electrons injected into the anode via EET are transferred to the cathode where oxygen reduction occurs, generating electricity. Thus, light to electrical energy conversion in BPVs is achieved on the basis of the functions of living photosynthetic microorganisms.

Microbial EET was originally identified and has been extensively studied in dissimilatory metal-reducing bacteria (DMRB) such as *Shewanella* spp. and *Geobacter* spp. (Lovley et al., 2004; Weber et al., 2006; Kato, 2015; Shi et al., 2016). DMRB are capable of utilizing solid-state Fe(III) (hydr)oxides as respiratory electron acceptors via EET in anaerobic environments, and redox-active proteins expressed in the outer membranes of these microbes are known to play an essential role in this process (Kato, 2015; Shi et al., 2016). Since Fe(II) ions are released to the environment as a consequence of EET to Fe(III) (hydr)oxides, EET from DMRB can have a significant impact on the Fe cycle in natural ecosystems (Lovley et al., 2004; Weber et al., 2006; Melton et al., 2014). In general, the capacity of DRMB to reduce metal oxides is closely correlated with the ability of the microbes to generate electricity (Bond and Lovley, 2003; Bretschger et al., 2007; Liu et al., 2010). Specifically, microorganisms that can reduce Fe(III) (hydr)oxides at a higher rate exhibit superior electricity generation characteristics, and *vice versa*. However, it is uncertain whether or not the (photo)electricity generation ability of photosynthetic microorganisms correlates with the capacity of such organisms to reduce Fe(III) (hydr)oxides. This is because the oxygen evolved as a consequence of photosynthetic activity rapidly oxidizes Fe(II) at pH values close to neutral, forming Fe(III) (hydr)oxides. Furthermore, none of the genes associated with EET in DMRB have been confirmed to appear in cyanobacteria (Kranzler et al., 2014).

The cyanobacterium *Synechocystis* sp. PCC 6803 (S6803) is an ideal model species for studying the correlation between the generation of (photo)electricity and the reduction of Fe(III) (hydr)oxides. S6803 is known to take up iron via the reduction of trace amounts of dissolved Fe(III) species using alternate respiratory terminal oxidase (ARTO) (Kranzler et al., 2011, 2014; Figure 1), and can grow using insoluble iron oxides as its sole iron source (Lamb et al., 2014). Furthermore, S6803 has been found to express electrically-conductive nanowires that are associated with the appearance of EET in DMRB (Gorby et al., 2006). Even so, there have been no attempts to quantitatively assess the ability of S6803 to reduce Fe(III) (hydr)oxides or to examine the correlation of such reduction with (photo)electricity generation. Therefore, the present work investigated the appearance of these phenomena in this microbe under a variety of conditions, such as by varying the light intensity and concentrations of oxygen and exogenous glucose. The aim of this study was to clarify the correlation between these two EET-related capabilities.

## Materials and Methods

### Bacterial Strains and Cell Culture Conditions

This work employed a glucose-tolerant, wild strain of S6803 (Williams, 1988; Shimakawa et al., 2014) that was grown and stored on solid BG-11 media plates (1.5% Bacto agar). Preculturing was performed by inoculating 30 mL portions of the liquid BG-11 medium in 100 mL flasks with cells from the agar plates. These flasks were then heated at 30°C with air bubbling under white light at an intensity of 20 μmol m^–2^s^–1^ to promote microbe growth. The main culture was obtained by inoculation of a 30 mL quantity of the BG11 medium to give an optical density of 0.02 at 730 nm (OD_730_), applying the same conditions as employed to produce the preculture.

### Electrochemical Analysis and Chlorophyll Fluorescence Measurements

The electrochemical measurements were carried out at 30°C in a single-chamber electrochemical cell with an indium-tin oxide (ITO) working electrode (3 cm^2^), an Ag/AgCl (saturated KCl) reference electrode and a platinum counter wire (Supplementary Figure 1). In preparation, S6803 cells were collected from the main culture with OD_730_ of 2–4 by centrifugation, washed twice with a 20 mM TES buffer (pH 7.5) and suspended in the same buffer. Following this, a 3.7 mL quantity of the TES buffer was added to the electrochemical cell, followed by the introduction of the cell suspension to the bottom of the cell to give an OD_730_ of 10. Situating the cells on the bottom working electrode was expected to increase the current obtained from this system. The generation of an anodic current was monitored using a potentiostat (HA-1510, Hokuto Denko, Tokyo, Japan) with the working electrode potential set at + 0.25 V relative to the reference electrode.

The chlorophyll fluorescence was assessed simultaneously using “e-PAM” set-up described previously (Beauzamy et al., 2020). Briefly, the signal collection fiber of dual PAM/Fiber fluorometer (Heintz Walz, Effeltrich, Germany) was positioned on the bottom ITO working electrode of the electrochemical cell (Supplementary Figure 1). Photosynthetic electron transport was driven by actinic light (635 nm), and the fluorescence signal (F) was originated from pulse-modulated measuring light (620 nm). For assessment of the fluorescence yield for cells adapted in dark conditions, the effective intensity of measuring light is set at very low level (Schreiber, 2004). The maximum fluorescence level obtained with fully reduced Q_*A*_ (Fm’), the primary quinone acceptor of photosystem (PS) II, was determined by applying 300 ms pulses of saturation light (7,000 μmol m^–2^ s^–1^). The effective quantum yield of the PSII [Y(II)] was calculated as (Fm’-F)/Fm’ (Baker, 2008).

### Fe(III) Reduction Assay

In preparation for these experiments, S6803 cells were collected by centrifugation, washed twice with Fe-free BG11 and suspended in the same medium such that the optical density at 730 nm was 5. Ferrihydrite was prepared following a process described in a previous publication (Lovley and Phillips, 1986) and added to the cell suspension to give a final concentration of 15 mM Fe, after which a portion of a sterilized stock solution of glucose was added. The cell suspension was subsequently incubated in darkness at 30°C under a N_2_ atmosphere with shaking, following which the concentration of Fe(II) was determined by the ferrozine method, as described previously (Lovley and Phillips, 1987). Briefly, the harvested cell suspension was mixed with an HCl solution to give a final concentration of 0.5 M. After incubation for 15 min, the suspension was centrifuged. A 10 μL aliquot of the resulting supernatant was added to 190 μL of a 50 mM HEPES buffer (pH 7.0) containing 2 mM ferrozine. The absorption of this solution at 562 nm was measured and the Fe(II) concentration was calculated according to a standard calibration curve prepared using known concentrations of FeSO_4_ in solution.

## Results

**FIGURE 2:**
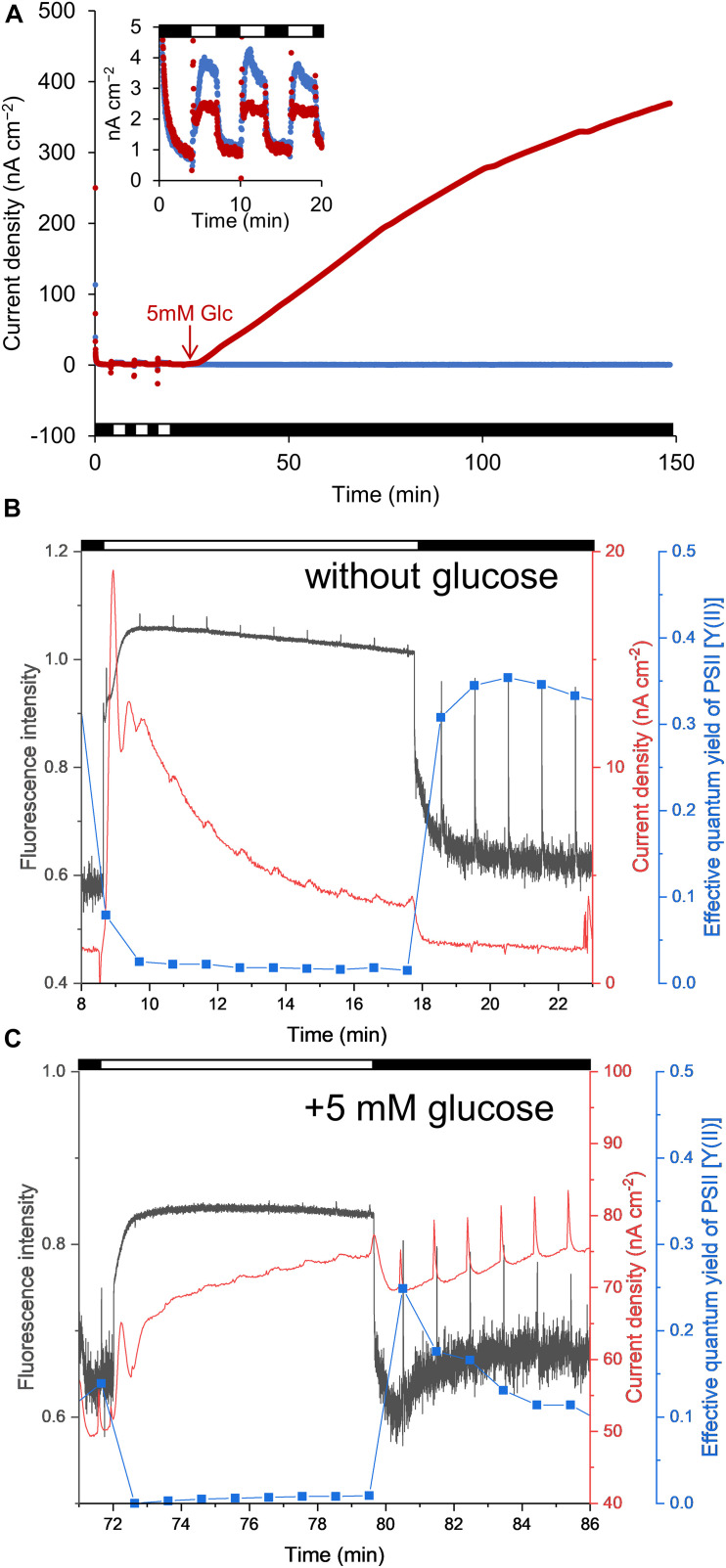
**(A)** Time courses of anodic current generation from S6803 cells at + 0.25 V vs. Ag/AgCl. Red and blue lines indicate the current with and without 5 mM glucose, respectively, added at the point indicated by the red arrow. The inset shows an enlarged view of the data up to 20 min. **(B,C)** Chlorophyll fluorescence (black lines), current (red lines) and effective quantum yield of the PSII complex [Y(II)] (blue lines) as functions of time with and without glucose. The spikes observed at every 1 min resulted from saturation pulses. The horizontal white and black bars in each graph indicate light [**(A)** 200 μmol m^–2^ s^–1^ **(B,C)** 260 μmol m^–2^ s^–1^] and dark conditions.

We initially measured the anodic current to ascertain the conditions under which the S6803 exhibited the capacity to reduce Fe(III) (hydr)oxides (Figure 1). The anodic current was found to slightly increase in response to light exposure under aerobic conditions, in agreement with previous reports (Figure 2A, inset) (McCormick et al., 2011; Cereda et al., 2014; Sekar et al., 2016; Wei et al., 2016; Saper et al., 2018; Zhang et al., 2018). When irradiation was ceased, the current sharply decreased. The current was also observed to gradually increase following the addition of glucose, while the current produced by the control sample without glucose was stable (Figure 2A). The anodic current was not observed in the presence of cells or in the presence of dead cells even when the glucose was added into the system (Supplementary Figure 2). These results clearly show that S6803 can generate EET current through dissimilation of glucose in darkness. Since the respiratory electron chain share some components with photosynthetic electron chain in S6803 cells (Lea-Smith et al., 2016; Figure 1), the effect of glucose addition can be assessed by simultaneously monitoring the chlorophyll fluorescence signal. In the absence of exogenous glucose, the chlorophyll fluorescence output decreased monotonically when the light was off while, in the presence of glucose, the fluorescence initially decreased and then increased after irradiation was ceased (Figures 2B,C, black lines). In addition, although the Y(II) in darkness was almost constant over time without glucose, this value decreased in the presence of glucose (Figures 2B,C, blue lines). The data acquired under the dark conditions indicate that exogenous glucose was metabolized in the S6803 cells, and that the respiratory electron transfer chain became reductive.

**FIGURE 3:**
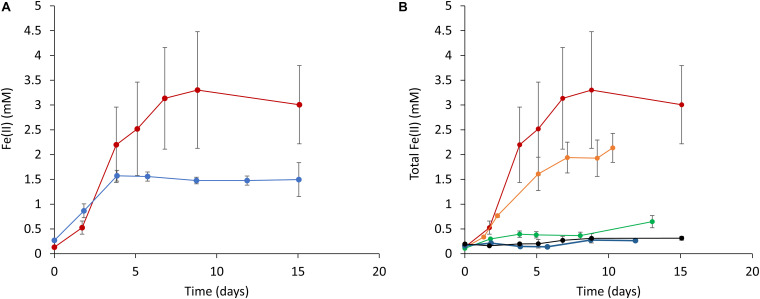
Time courses of ferrihydrite reduction determined using a ferrozine assay. **(A)** A comparison of ferrihydrite reduction under anaerobic (red line) and aerobic (blue line) conditions. **(B)** The effect of glucose concentration on ferrihydrite reduction by S6803 cells. Red, orange, green, blue, and black lines indicate initial glucose concentrations of 15, 5, 3, 1, and 0 mM, respectively. Red lines in **(A,B)** are identical data. Values are means ± one standard deviation (error bars) of three replicate trials.

Based on the above results, it was expected that the cyanobacterial reduction of Fe(III) (hydr)oxides would occur to the greatest extent under dark conditions in the presence of glucose. Therefore, we subsequently attempted to quantitatively investigate the effect of glucose on ferrihydrite reduction in the absence of light. Figure 3 plots Fe(II) concentrations over time (as determined by the ferrozine assay) in cell suspensions containing ferrihydrite. Increases in the Fe(II) concentration are evident in those samples incubated under both aerobic and anaerobic conditions (Figure 3A). Notably, ferrihydrite reduction was also observed in a batch of fresh BG-11 medium containing ferrihydrite and glucose but not S6803 cells (Supplementary Figure 3). In addition, when a cell suspension incubated with glucose but without ferrihydrite was mixed with ferrihydrite just prior to the ferrozine assay, the reduction of ferrihydrite did not occur (Supplementary Figure 4). This result demonstrates that the Fe(II) increase in Figure 3A did not result from ferrihydrite reduction by intracellular reducing equivalents. Thus, the above data indicate that S6803 was able to reduce extracellular solid-state ferrihydrite in the presence of glucose.

The effect of the glucose concentration on the ability of the S6803 to reduce ferrihydrite under dark conditions was subsequently examined. Because the end amount of reduced ferrihydrite in samples incubated under aerobic conditions was lower than that under anaerobic conditions (Figure 3A), the effect of glucose was examined under anaerobic conditions. Trials were performed by adding 15 mM ferrihydrite, S6803 cells and glucose to BG-11 medium followed by incubation under anaerobic conditions, and higher glucose concentrations were found to promote ferrihydrite reduction (Figure 3B). The chlorophyll concentration during incubation remained relatively constant, and so it is probable that the S6803 cells maintained their cellular components during these experiments even under continuous anaerobic, dark conditions (Supplementary Figure 5). These results establish that the cyanobacterial ferrihydrite reduction process was affected by the exogenous glucose concentration. In the case that a suspension of cells containing 5 mM glucose was exposed to light, ferrihydrite reduction under aerobic conditions was found to be slower than that under anaerobic dark conditions, although the rate of the reductive generation of Fe(II) was greater than that observed in a control experiment without cells (Supplementary Figure 3). These results suggest that cyanobacteria can reduce insoluble Fe(III) (hydr)oxides even in aerobic environments.

**FIGURE 4:**
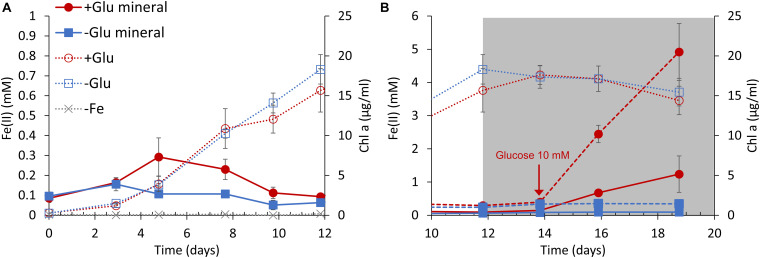
S6803 and Fe(II) concentrations over time under anaerobic conditions with ferrihydrite as the sole iron source. The S6803 cells were inoculated to give an optical density at 730 nm of 0.1 and grown with N_2_ bubbling. **(A)** The rapid growth phase under 100 μmol m^–2^ s^–1^ light irradiation. **(B)** The stationary phase under dim light irradiation (gray background). The red and blue lines indicate data obtained with and without glucose, respectively. The solid and broken lines indicate the Fe(II) concentrations in cell suspension and solid iron samples (see also Supplementary Figure 8), respectively. The dotted lines indicate chlorophyll concentration and the gray dotted lines indicate chlorophyll concentration in cultures without ferrihydrite. Values are means ± one standard deviation (error bars) of three replicate trials.

Based on the above results, the growth of S6803 cells was measured in the presence of both ferrihydrite and glucose under anaerobic conditions. Cells were grown under light irradiation at an intensity of 100 μmol m^–2^ s^–1^ while oxygen was continuously removed by nitrogen bubbling. During these trials, ferrihydrite was added to the BG11 medium as the sole iron source. Cells in cultures without ferrihydrite did not grow, as expected (Figure 4A, gray line) while, in contrast, cells in cultures with ferrihydrite grew well under anaerobic conditions (Figure 4A, red and blue dotted lines). In agreement with a previous report that S6803 can grow using insoluble iron oxides as the sole iron source (Lamb et al., 2014), S6803 was found to be capable of utilizing ferrihydrite as its iron source even under anaerobic conditions. Although the growth rate was unaffected by the addition of 5 mM glucose, the amount of Fe(II) slightly increased in the early stage of culturing in the presence of glucose (Figure 4A, red solid line). After the light intensity was decreased to 5 μmol m^–2^s^–1^ to mimic dim light conditions in a typical internal microbial ecosystem (Fourçans et al., 2004; Ciani et al., 2005; Ohkubo and Miyashita, 2017), the chlorophyll concentration remained almost constant both with and without glucose (Figure 4B, red and blue dotted lines). Following this transition to dim light conditions, the glucose concentration was increased to 10 mM, causing a rapid increase in the amount of Fe(II) (Figure 4B, red broken and solid lines). The above results suggest that ferrihydrite reduction sufficient for iron acquisition and cell growth (Kranzler et al., 2011, 2014; Lamb et al., 2014) is possible without exogenous glucose, although glucose can promote ferrihydrite reduction to generate an excess of Fe(II) for S6803 growth. In fact, the ferrihydrite reduction rates obtained with 3–15 mM glucose were about 100 times larger than the value reported for dissolved Fe(III) species without glucose (Kranzler et al., 2014; Supplementary Figure 6).

## Discussion

In contrast to the light-dependent current increases reported elsewhere, the current in the present work was found to increase under dark conditions upon the addition of glucose. This result clearly shows that the EET current in S6803 can be also generated through dissimilation of glucose. Details of mutual relationship between the light-dependent and glucose-dependent EET pathways remains unknown. However, given that the photosynthetic and respiratory electron transport share some components in S6803 (Lea-Smith et al., 2016), it is reasonable to consider that the light-dependent and glucose-dependent EET pathways are also mutually correlated each other, as schematically shown in Figure 1. In fact, it was reported that respiratory systems and NADPH are known to participate in the electron pathways of photocurrent generation (Bombelli et al., 2011; Bradley et al., 2013; Saper et al., 2018). The molecular mechanisms that occur in conjunction with EET to Fe(III) (hydr)oxides in DMRB have been well investigated, and it is now known that outer membrane cytochromes play an essential role (Weber et al., 2006; Kato, 2015; Shi et al., 2016). However, these redox-active proteins have not yet been identified in S6803. It has been suggested that ARTO and/or Type IV pili are involved in the iron uptake process in S6803 (Kranzler et al., 2014; Lamb et al., 2014), and such factors may also contribute to ferrihydrite reduction and electricity generation by this microbe (Figure 1) [of note, a recent paper reported that pili has little impact on the photo-current generation capability (Thirumurthy et al., 2020)]. Another possibility is that a diffusive electron mediator excreted from the cells was responsible for ferrihydrite reduction. In fact, some redox peaks were observed in cyclic voltammograms obtained from these cells (Supplementary Figure 7). These peaks are consistent with previous reports and are attributed to small redox-active metabolites related to EET capability (Saper et al., 2018; Zhang et al., 2018).

It is also helpful to examine the possible effects of cyanobacterial ferrihydrite reduction on microbial ecosystems. Since cyanobacterial habitats become aerobic as a result of oxygenic photosynthesis, cyanobacteria have been regarded as oxidizers of Fe(II) in natural environments. However, the present study demonstrates that cyanobacteria can also act as reducers of Fe(III). In fact, Fe(II) has been detected in aerobic regions within sediment, microbial mats and seawater where cyanobacteria are also found (O’Sullivan et al., 1991; Wieland et al., 2005; Rentz et al., 2007). Although a certain amount of Fe(II) can be generated abiotically by photo-generated superoxides or photo-reaction with Fe(III)-binding ligands (Voelker and Sedlak, 1995; Barbeau, 2006), the present work suggests that Fe(II) can also be produced by EET associated with photosynthetic microorganisms in environments where glucose is present. Microbial mats are one such ecosystem, and *Synechocystis* species are often found in microbial mats (Fourçans et al., 2004; Singh et al., 2011) where they produce a polysaccharide as an extracellular polymeric substance (EPS) (Flemming and Wingender, 2010; Stuart et al., 2016). It has been estimated that the concentration of carbohydrates in this EPS is 45 mg/g on a wet basis, and that glucose residues account for almost all the carbohydrate (Stuart et al., 2016). This amount therefore corresponds to approximately 250 mM glucose. The present study revealed that ferrihydrite reduction occurred in the presence of glucose at a concentration of only 3–15 mM (Figure 3). Together, these data suggest that cyanobacterial Fe(III) reduction could be promoted by exogenous glucose even in natural microbial mats.

It is also of interest to compare the Fe(II) concentration range observed in our laboratory experiments with those found in natural environments. In iron-rich microbial mats containing cyanobacteria, Fe(II) has been found at concentrations from 1 to 10 mM even in the aerobic top 2.0 mm of the mats (Wieland et al., 2005). In our experiments, the maximum concentration of Fe(II) generated by the S6803 cells in the absence of light was comparable to the level in the aerobic layers of such iron-rich microbial mats (Figure 3B). Thus, the cyanobacterial reduction of insoluble Fe(III) may help maintain the concentration of Fe(II) in the photic layers of microbial mats (Supplementary Figure 8).

Although soluble Fe(II) is typically almost completely oxidized by abiotic reactions with oxygen under aerobic conditions, the Fe(II) concentration was increased and then maintained by S6803 cells even under aerobic dark conditions (Figure 3A). There are two possible explanations for this result. Firstly, the ferrihydrite reduction rate may have been faster than the abiotic Fe(II) oxidation rate and, secondly, the Fe(II) that was generated could have been poorly reactive with oxygen. The oxidation rate of dissolved Fe(II) at a concentration of 1 mM in a pH 7 solution under ambient air is estimated to be 9.6 mM h^–1^ (Rentz et al., 2007), which is much faster than the rate of reduction by the S6803 cells, indicating that the first possibility can be excluded. Although we tried to confirm the presence of Fe(II) in solid phase by X-ray diffraction (XRD) and transmission electron microscope (TEM), characteristic signal for crystallization of Fe(II) could not observed due to small particle size (<5 nm) (Supplementary Figure 9). However, an increase in Fe(II) in the solid fraction was observed during the ferrihydrite reduction experiments (Supplementary Figure 10). Thus, it is likely that Fe(II) was protected from oxygen in the solid phase and thus stabilized.

In summary, the present work demonstrated that S6803 can reduce ferrihydrite via EET. Both the reduction rate of ferrihydrite and the microbial current were higher under dark conditions in the presence of glucose than under light conditions. Thus, the accumulation of organic substances in local environments surrounding photosynthetic microbes could be important in terms of the enhanced reduction of iron (hydr)oxides and the generation of microbial electricity. We anticipate that further studies along these lines will provide a deeper understanding of the biogeochemical cycling of iron in nature and will also assist in the development of biophotovoltaics.

## Data Availability Statement

The original contributions presented in the study are included in the article/Supplementary Material, further inquiries can be directed to the corresponding author/s.

## Author Contributions

KT, GS, and SN designed the research. KT, GS, and TH performed the research. KT, GS, TH, ShK, SoK and SN analyzed the data. KT, GS and SN wrote the manuscript. All authors contributed to the article and approved the submitted version.

## Conflict of Interest

The authors declare that the research was conducted in the absence of any commercial or financial relationships that could be construed as a potential conflict of interest.
