# Supplementary material for: Ferrihydrite Reduction by Photosynthetic Synechocystis sp. PCC 6803 and Its Correlation With Electricity Generation
Source: Front Microbiol. 2021 Mar 8;12:650832. doi: 10.3389/fmicb.2021.650832 (PMC7982531; doi:10.3389/fmicb.2021.650832)
Supplement: Supplementary file 1 [file Data_Sheet_1.docx]

Supplementary Material

**SI methods**

**Characterization of solid products**

Solid ferrihydrite particles were harvested from sample suspension containing S6803 cells as described in Fig. S8C. Briefly, ferrihydrite particles and S6803 cells were harvested by centrifugation at 12,000 ×g for 5 min, followed by removal of supernatant. S6803 cells in the pellet were disrupted by adding equal amount of methanol as initial sample volume. The suspension was centrifuged again, and the pellet was washed by adding 10 mL water. After the centrifugation, the washed pellet was dried in vacuum centrifugal concentrator (CC-105 TOMY, Tokyo, Japan). The dried pellet was analyzed by X-ray diffraction (XRD) and transmission electron microscope (TEM). XRD patterns recorded on a PANalytical X'Pert PRO diffractometer using Cu-Kα radiation. TEM images were taken on a Hitachi H-9000NAR instrument operated at 300 kV.


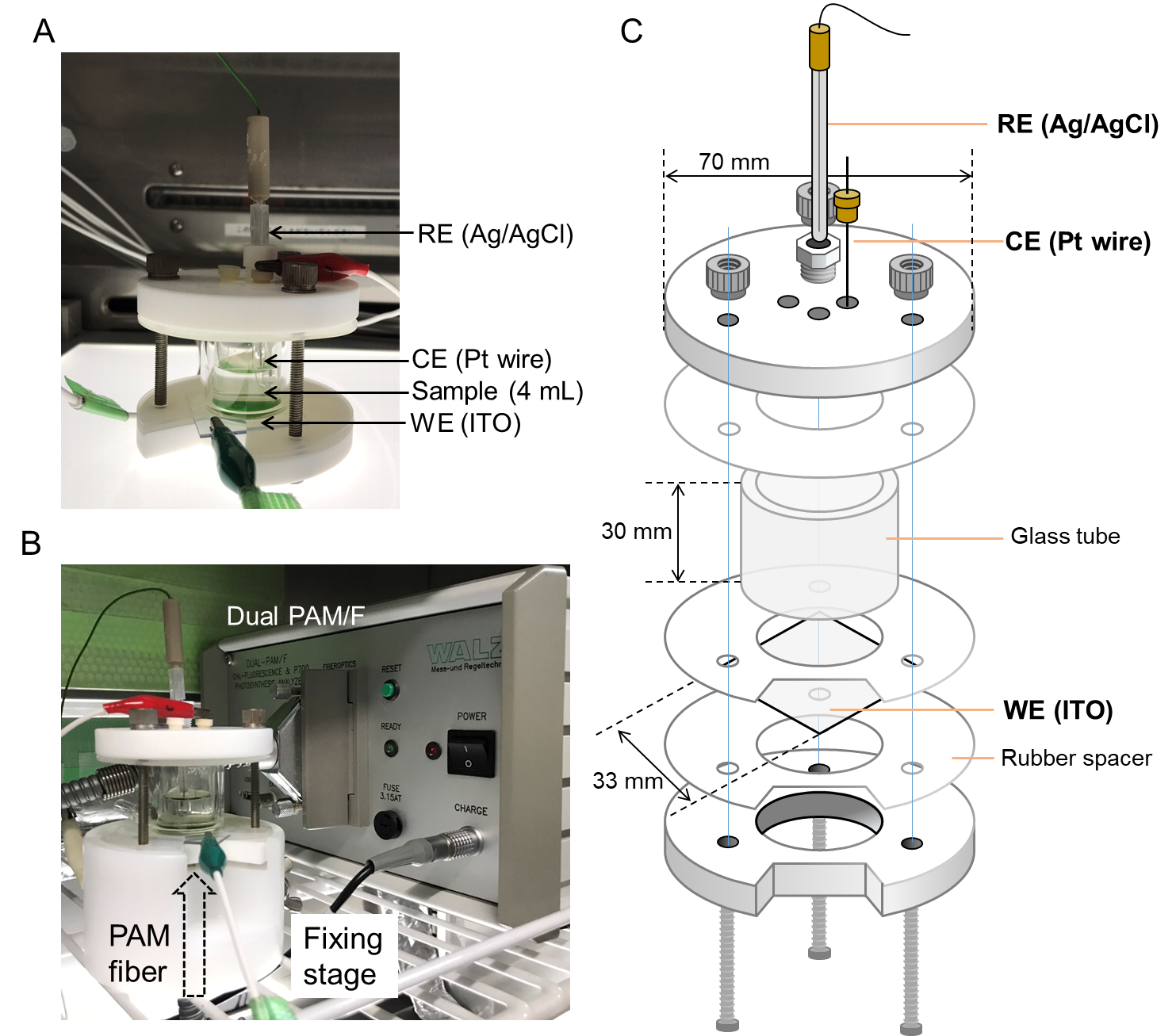


**Figure S1.** Pictures of set-ups of (A) electrochemical and (B) simultaneous chlorophyll fluorescence measurements. (C) Schematic of the electrochemical reactor. CE; counter electrode, RE; reference electrode, WE; working electrode.


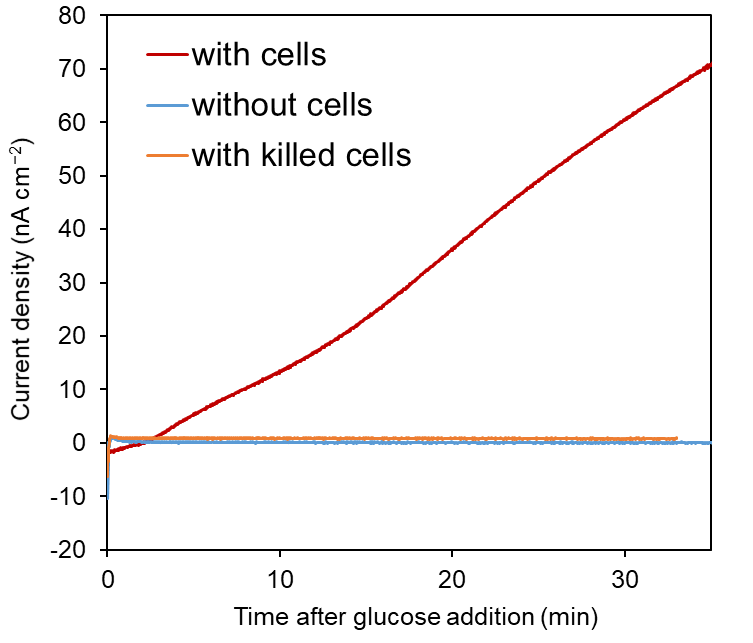


**Figure S2.** Increase of anodic current by addition of 5 mM glucose was observed only in the presence of living S6803 cells. Control sample without cell or with dead cells prepared by autoclave treatment (121 ºC, 20 min) did not produce anodic current by addition of glucose.


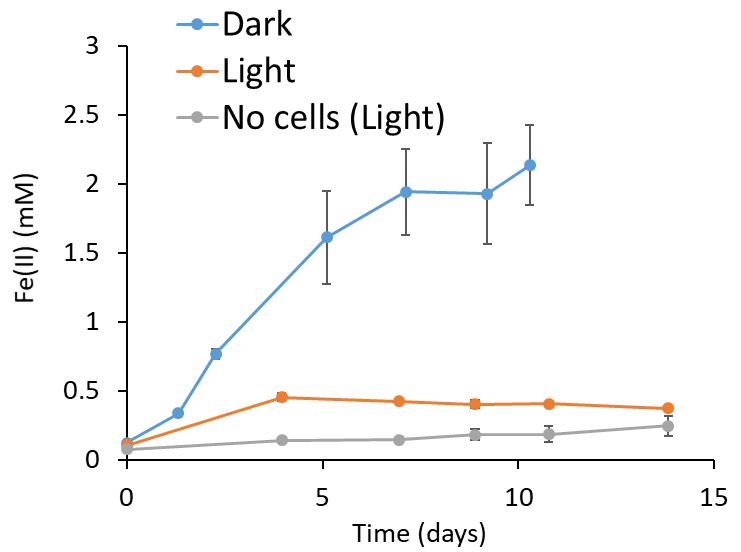


**Figure S3.** Time course of Fe(II) concentration. 15 mM ferrihydrite was incubated with 5 mM glucose and S6803 cells under dark condition (blue line), 100 μmol m^−2^ s^−1^ light irradiation (orange). For control sample, 15 mM ferrihydrite was incubated with 5 mM glucose without S6803 cells under light condition (gray line). Values are means ± SD (bars) of three biological replicates.


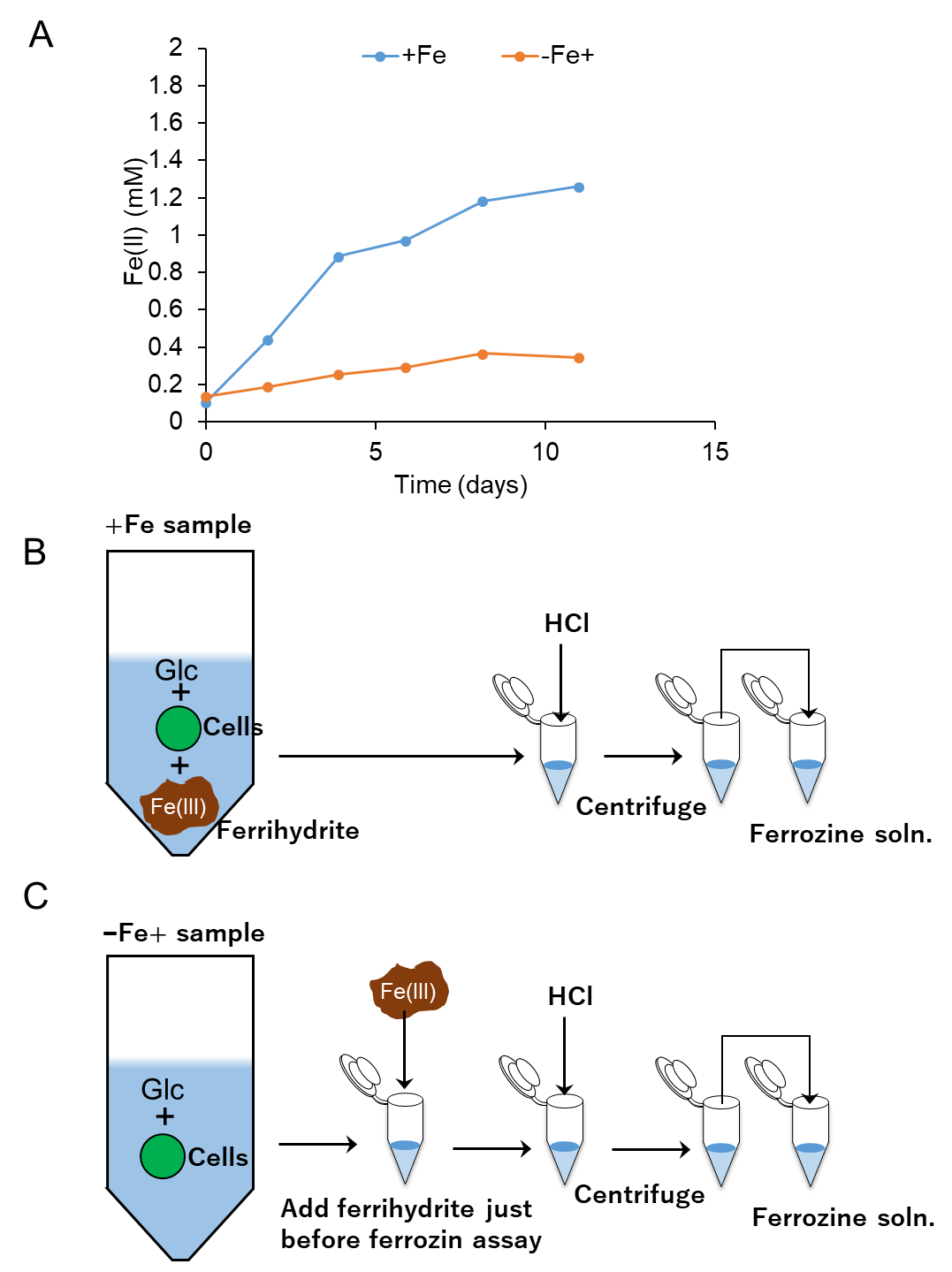


**Figure S4.** Time course of Fe(II) concentration and schematic explanation of samples. (A) 5 mM glucose and S6803 cells were incubated with (blue line) or without (orange line) 15 mM ferrihydrite. Fe(II) concentration in the sample indicated as blue line in (A) was measured by standard ferrozine assay as described in (B). For samples indicated orange line in (A), 15 mM ferrihydrite was added just before the standard ferrozine assay as described in (C) to verify whether cellular reducing equivalents reduce ferrihydrite during ferrozine assay.


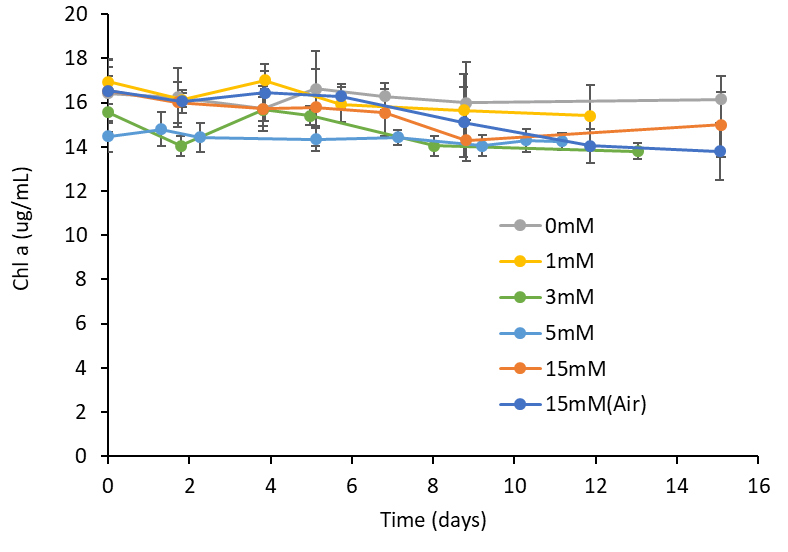


**Figure S5.** Time course of chlorophyll concentration during the ferrihydrite reduction assay in Fig. 3. Values are means ± SD (bars) of three biological replicates.


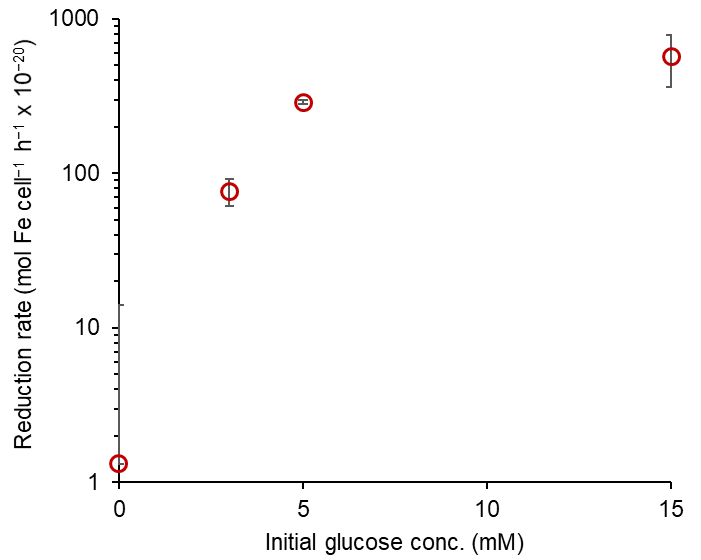


**Figure S6.** Fe(II) generation rate was calculated using data shown in Fig. 3 for first 4 days. Values are means ± SD (bars) of three biological replicates.


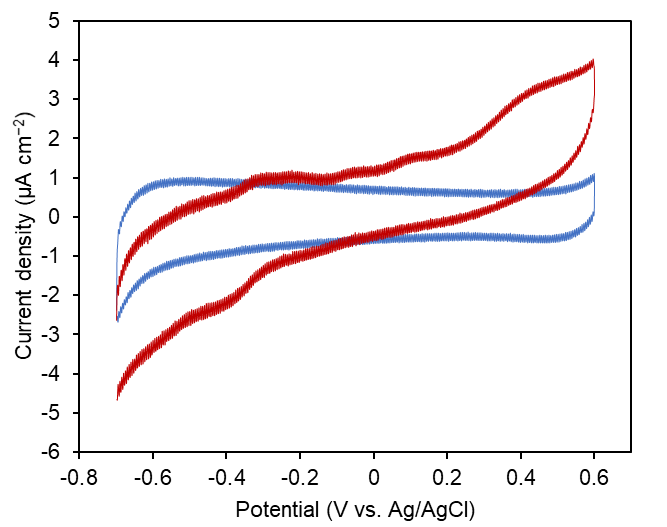


**Figure S7.** Cyclic voltammograms of S6803 cell suspension measured after current generation experiment with (red line) and without (blue line)5 mM glucose shown in Fig. 2A. Scan rate was 50 mV s^−1^.


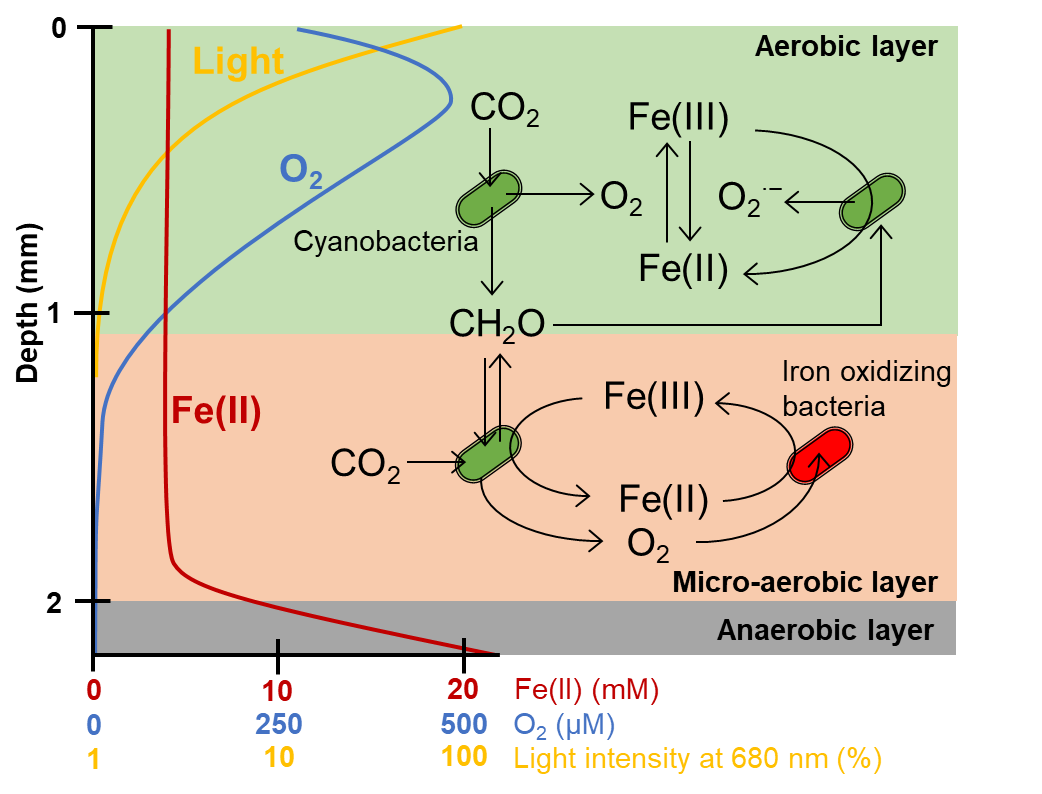


**Figure S8.** Schematic of proposed iron redox cycle related to cyanobacteria. Fe(II) concentration, oxygen concentration, and light intensity are indicated by red, blue and yellow lines based on previous works (For Fe(II) and O_2_, Wieland et al. 2005; for light intensity, Fourcans et al. 2004).


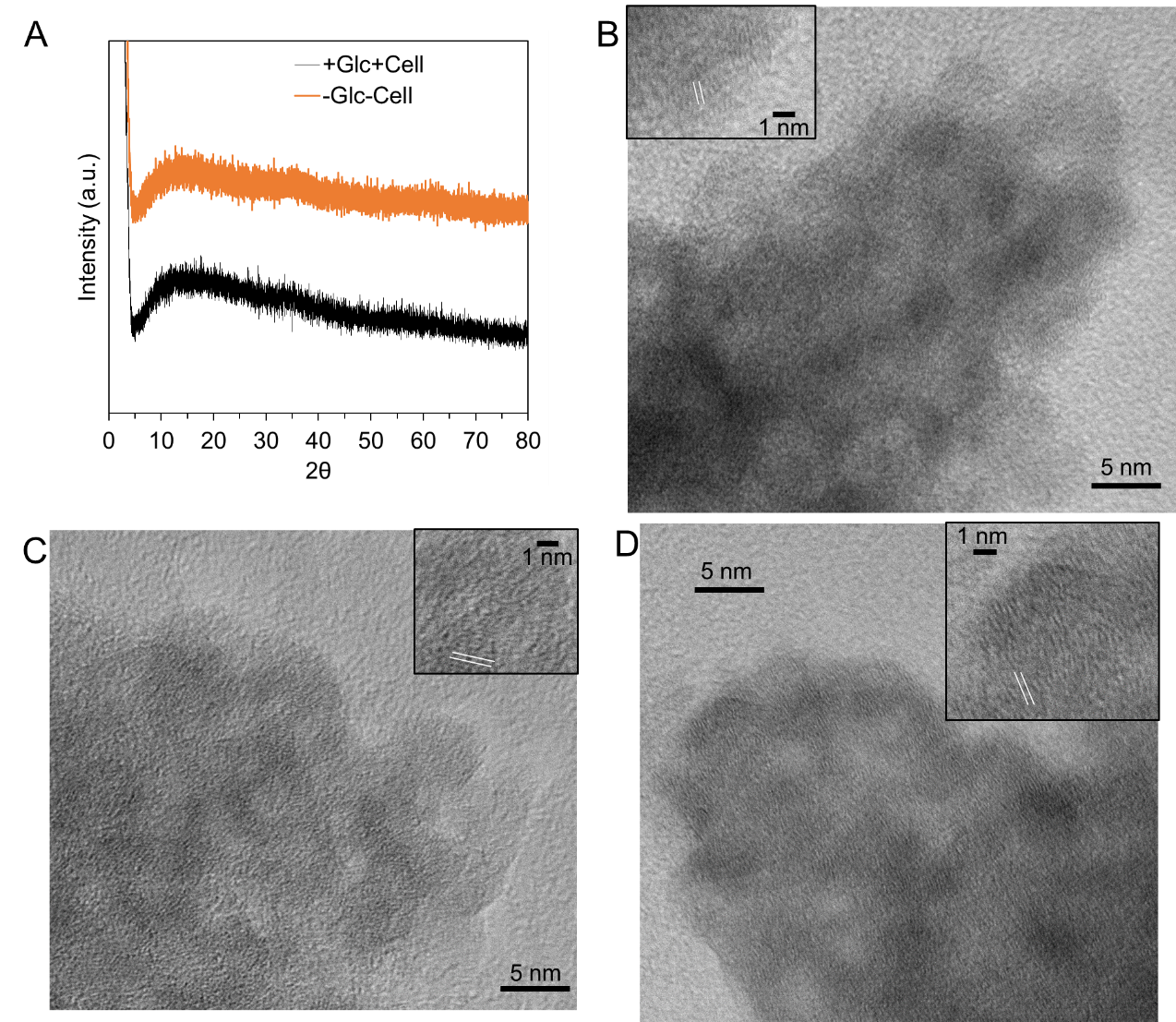


**Figure S9.** Characterization for ferrihydrite particles. (A) XRD patterns for synthesized ferrihydrite (orange line) and products of ferrihydrite reduction by S6803 (black line). (B-D) TEM images of synthesized ferrihydrite (B), products of the sample incubated with S6803 cells but without glucose (C), and products of the sample incubated with both S6803 cells and glucose (D) showed ferrihydrite particles were smaller than 5 nm. White lines in insets of TEM images indicate representative lattice fringes. Although lattice fringes were observed in the TEM images, no significant peak was confirmed in the XRD pattern. This is likely to be because small particle size limits the repeating arrangements of lattice structures, leading to small wave interference effect. This small size effect makes it difficult to characterize solid Fe(II) species generated by S6803 cells.


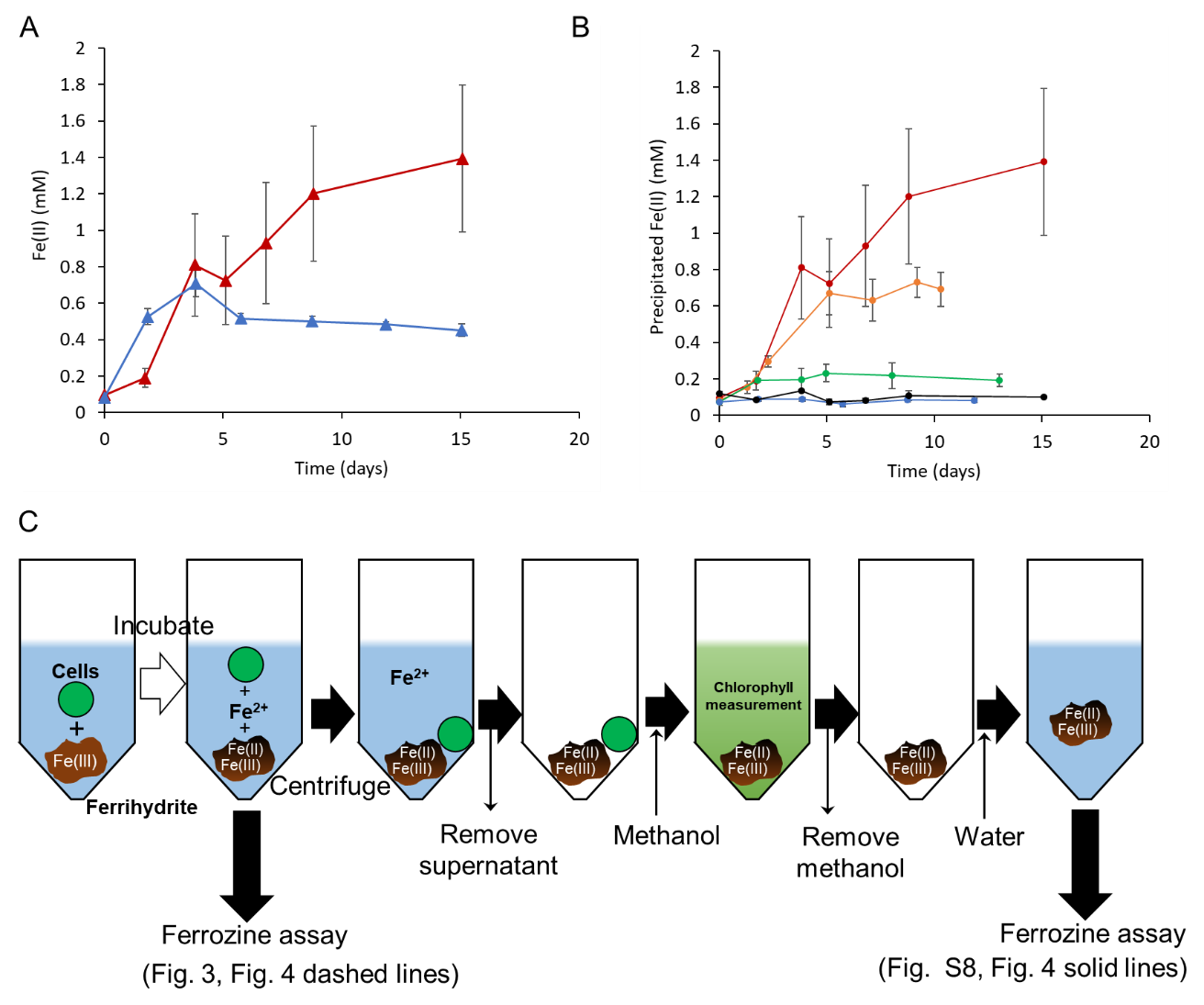


**Figure S10.** (A,B) Time course of Fe(II) concentration of solid phase and (C) schematic explanation of sample preparation. For determining Fe(II) involved in solid phase, supernatant and S6803 cells were removed from the ferrihydrite suspension as described in (C). (A) Comparison of Fe(II) concentration in solid phase under anaerobic (red line) and aerobic conditions (blue line). (B) Dependency of Fe(II) concentration in solid phase on glucose concentration. Red, orange, green, blue and black lines indicate initial glucose concentration of 15, 5, 3, 1, 0 mM, respectively. Values are means ± SD (bars) of three biological replicates.
